# Effect of Yiqi Huoxue Granules on Platelet Activation Induced by Thrombin

**DOI:** 10.1155/2021/6622848

**Published:** 2021-07-16

**Authors:** Zhen Lei, Shuibo Gao, Xinzhou Wang, Haixia Gao, Yongjun Han, Zhentao Wang, Hong Wu

**Affiliations:** ^1^Central Laboratory, Henan Province Hospital of Traditional Chinese Medicine, The Second Affiliated Hospital of Henan University of Chinese Medicine, Zhengzhou 450002, China; ^2^Laboratory of Cell Imaging, Henan University of Chinese Medicine, Zhengzhou 450002, China; ^3^Institute of Cardiovascular Disease, Henan University of Chinese Medicine, Zhengzhou 450002, China

## Abstract

**Objective:**

To study the effects of Yiqi Huoxue (YQHX) granules on platelet activation and aggregation induced by thrombin.

**Methods:**

The effect of YQHX on platelet aggregation rate was detected by platelet aggregation instrument; the effect of YQHX on thrombosis time was observed by the mouse mesentery thrombosis model. DAMI cells were induced to transform into platelet-like granules using PMA, and the effects of SCH (PAR-1 inhibitor) on thrombin-induced changes in platelet intracellular calcium concentration, PAR-1 protein expression, and phosphorylation of MAPK were examined.

**Results:**

Compared with the control group, the platelet aggregation rate, PAR-1 protein expression, phosphorylation of ERK1/2, and p38 protein in the YQHX group decreased (*P* < 0.05), and there was no significant difference between the YQHX + SCH group and YQHX group (*P* > 0.05).

**Conclusion:**

YQHX suppresses the platelet activation induced by thrombin by inhibiting PAR-1 expression.

## 1. Introduction

Percutaneous coronary intervention (PCI) is the main treatment for acute coronary syndrome, but after the treatment, the patients tend to suffer from secondary damage of myocardia tissues triggered by pathological change such as microthrombus [1]. Platelet activation plays a critical role in thrombosis [2]. As an effective platelet activator, thrombin (THR) induces platelet aggregation via protease-activated receptors (PARs) [3]. PAR-1 is the main receptor in the platelet membrane, and under the induction of THR, it can be coupled with the G12/13, Gq, and Gi proteins, which will activate the intracellular signaling pathways such as mitogen-activated protein kinase (MAPK), thus triggering reactions such as platelet aggregation [4, 5]. Inhibition of PAR-1 expression can help reduce artery thrombosis, but it does not affect the bleeding time or coagulation characteristics [6]. Vorapaxar is a PAR-1 receptor inhibitor drug, which is currently at its phase 3 clinical trial. Vorapaxar can inhibit platelet aggregation and reduce the mortality of cardiovascular diseases, but the risk of intracranial hemorrhage is increased by three times [7]. Therefore, a popular research subject is to seek new anticoagulation and antithrombotic drugs with less side effects. The Yiqi Huoxue granule (YQHX) is made of *Panax ginseng*, *Astragalus membranaceus*, *Radix paeoniae rubra*, and *Stigma croci*, which has the effects of benefiting qi for activating blood circulation as well as clearing and activating the channels and collaterals. YQHX can inhibit platelet aggregation, which can also improve the cardiac function of ACS patients, alleviate their clinical symptoms, and lower the mortality rate [8]. The results of preliminary study show that YQHX can reduce the endothelial inflammation level, weaken the damage of myocardia cells caused by oxidative stress [9], and inhibit the platelet aggregation and thrombosis induced by ADP under the influence of TXB2 [10]. However, it is still unclear whether YQHX can inhibit the change of platelet activation function triggered by thrombin via the PAR-1 receptor. This study aims to investigate the influence of YQHX on the platelet activation function triggered by thrombin.

## 2. Materials and Methods

### 2.1. Experimental Drug

YQHX is made of *Panax ginseng*, *Astragalus membranaceus*, *Radix paeoniae rubra*, and *Stigma croci* granules in the proportions of 3 : 6:3 : 2, and the granules were bought from Sichuan Neo-Green Pharmaceutical Technology Development Co., Ltd. The batch numbers of various single Chinese herb granules are as follows: *Panax ginseng* (No. 17010055), *Astragalus membranaceus* (No. 17010092), *Radix paeoniae rubra* (No. 16060138), and *Stigma croci* (No. 16080067). 25 mL normal saline was added to 10 g YQHX granule, and the concentration was 0.4 g/mL after dissolution. According to the body surface area conversion coefficient between human and mouse, the administration dosage of 12.9 mg/100 g/d was obtained.

### 2.2. Test of Platelet Aggregation Rate

This study was conducted under the approval and supervision of the Ethics Committee of the Second Clinical Medical School of Henan University of Chinese Medicine. The 10 health volunteers participating in the study had signed the informed consent and agreed to have their blood samples used in our study. By referring to [11], blood was drawn from the elbow vein of participants and then put into the vacuum tube containing 1 : 9 (v/v) 3.8% sodium citrate, and 300 g sample was centrifuged for 15 min to separate plasma containing rich platelets. 200 *μ*L PRP was put into a glass tube, the magnetic rotor was added, and then 25 *μ*L of YQHX granules in different concentrations (0.05, 0.1, and 0.2 mg/mL) was added for incubation for 10 min under 37°C. In the control group, the normal saline was added, then 0.25 U/mL thrombin (Sigma-Aldrich, USA) was added, and it was stirred for 5 min under 37°C. By using the aggregometer (Helena, USA), the platelet aggregation process and maximum aggregation rate were recorded according to the transmittance, and the inhibition rate of platelet aggregation was calculated. By using the same method, the final 800 g was centrifuged for 5 min to collect the platelets to be used in the follow-up western blot test.

### 2.3. Assay of Mesenteric Artery Thrombosis Induced by FeCl_3_

The C57BL/6 mice were bought from Beijing Vital River Laboratory Animal Technology Co., Ltd. (certificate no. 1100111911045302) and fed in the Experimental Animal Center of Henan Province Hospital of TCM (SYXK (Yu) 2016–0009), with Animal Ethics Certificate no. 20180316WZ. The male C57BL/6 mice of 8–10 weeks old were randomly divided into 3 groups, each group including 10 mice. Two groups were administered with aspirin (30 mg/k) and YQHX (12.9 mg/100 g), respectively, and the control group was administered with normal saline of the same amount. The mice were administered for 7 consecutive days, and test was conducted 2 h after the last administration. The in vivo mesenteric arterial thrombosis experiment was conducted according to [12]. The mice were anesthetized using urethane and had tail vein injection of 6G 0.05% rhodamine (20 *μ*L/10 g). Then, a 1 cm incision was cut along ventrimeson, the small intestine was gently squeezed out and put into the Petri dish, and a swab was used to spread the intestine out. Thrombosis was induced, the 1-2 mm filter paper soaked with 4% FeCl_3_ solution was used to cover the mesenteric artery for 2 min, and then it was rinsed with normal saline. It was placed upside down under the fluorescence microscope (Nikon, Japan) to observe for 40 min, and the mesenteric artery thrombosis was recorded.

### 2.4. Cell Culture

Since the platelet is akaryote, during preliminary study in the lab, PMA was used to induce the DAMI megakaryocytes to convert into the platelet-like granules, and then the platelet activation was measured [11, 12]. The DAMI cells were bought from Shanghai Cell Bank. The DAMI cells were suspended in the RPMI 1640 culture medium (Sigma, USA), and this culture medium contained 10% inactivated fetal calf serum and 100 U/mL cynomycin/streptomycin antibiotics. The cells were cultured under the temperature of 37°C, 5% CO_2_ and saturated humidity. Cells were passaged when the cell density exceeded 90%. Before being inoculated to the culture plate, the cell concentration was adjusted to 1.0 × 10^5^ cells/mL. 10 nM phorbol myristate acetate (PMA for short) (MedChemExpress, USA) was used to induce the DAMI cells to differentiate the platelet granules in 4 days. On the third day of induction, the YQHX granule (0.2 mg/mL) was added to have intervention for 24 h; on the fourth day, the PAR-1 inhibitor SCH79797 (1 *μ*M) (Abcam, UK) was added to have intervention for 1 h, then thrombin (1 U/mL) was added, and after reaction for 5 min, cells were collected to conduct intracellular calcium level and western blot tests.

### 2.5. Intracellular Calcium Level Test

After intervention with the method described in Section 2.4, the cellular supernatant was abandoned after centrifugation. The cells were rinsed for 3 times using the HEPES solution, the coloring agent FLUO 4-AM (MedChemExpress, USA) with final concentration of 5 *μ*M was added to fully mix, and, then, it was incubated for 30 min away from light under 37°C. After incubation, it was rinsed with HEPES twice. Then a certain amount of HEPES was added; it was place upside down on the fluorescence microscope stage, and it was observed using the excitation wavelength of 494 nm and emission wavelength of 516 nm. After the assay started, the calciferous thromboplastin was added, and the calcium imaging system (ANDOR, UK) was used to record the change of fluorescence intensity. The captured images were processed using the Andor iQ2 image analysis software, and the area under the curve (AUC) after induction with agonist was the change of intracellular Ca^2+^ concentration in platelets.

### 2.6. Western Blot Test

The collected platelet or cell sample was added with strong RIPA lysate (Wuhan Boster Biological Technology Co., Ltd., China) protein (including the phosphoric acid protease inhibitor and PMSF protease inhibitor); then, it was oscillated intensely for 5 min, and it had lysis on ice for 1 h. Next, it was centrifuged for 5 min under 12000 r/min, and the supernatant was collected, which was the total protein. The BCA Protein Quantitation Kit (Shanghai Beyotime Biotechnology Company, China) was used to measure the protein concentration, and after dilution, the 4x SDS loading buffer was used to boil and process the sample. The SDS-PAGE protein electrophoresis was conducted, and then, the protein was transferred to the PVDF membrane (Millipore, USA). It was added with 5% skim milk and sealed under 37°C for 2 h. Next, the primary antibodies were added for overnight incubation under 4°C (antibodies GAPDH, ERK1/2, P38, JNK, and PAR-1, and phosphorylated antibodies ERK1/2, P38, and JNK). After it was thoroughly washed using TBST, second antibodies were added for incubation under 37°C for 1 h. Then, the ECL chemiluminescence reagent (Beyotime, China) was used for color development, and the chemiluminescence imaging system (Bio-Rad, USA) was used to collect and analyze the images.

### 2.7. Statistical Processing

The IBM SPSS Statistics 21.0 statistical software was used. The measurement data were represented as $\overset{－}{x}$ ± *s*. One-way analysis of variance was adopted for comparison among groups, and the LSD method was used for comparison between any two groups. *P* < 0.05 indicates significant difference in statistics.

## 3. Results

### 3.1. Influence of Different Concentrations of YQHX Granules on the Platelet Aggregation Rate Induced by THR

**Figure 1:**
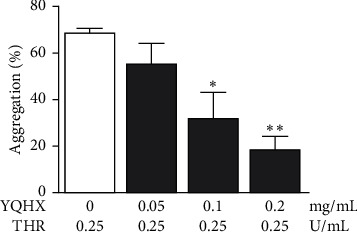
Comparison of platelet aggregation rates among various groups. *Note.* Compared with the control group, ^*∗*^*p* < 0.05 and ^*∗∗*^*p* < 0.01; *n* = 6.

The results are as shown in Figure 1. In the control group, the platelet aggregation rate induced by THR was 68.67 ± 2.03%. Compared with the control group, the platelet aggregation rate of YQHX granule (0.1 mg/mL) intervention group was 32.00 ± 11.37% (*P* < 0.05), and the platelet aggregation rate of YQHX granule (0.2 mg/mL) intervention group was 18.67 ± 5.78% (*P* < 0.01), which indicates that the platelet aggregation rate decreased with the increase in the concentration of YQHX granule.

### 3.2. Influence of YQHX Granules on the Mesenteric Artery Thrombosis Time of Mice

**Figure 2:**
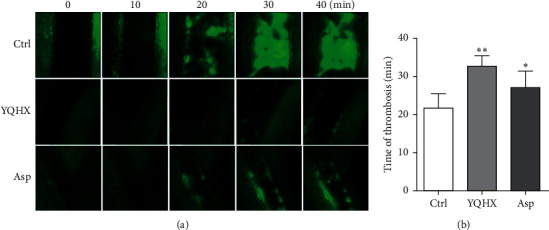
Comparison of the mesenteric artery thrombosis time of mice among various groups. (a) Comparison of thrombosis under fluorescence microscope after the mesenteric artery was damaged by FeCl3 among the control group (Ctrl), YQHX group (YQHX), and aspirin group (Asp), and the green granules are platelets; (b) mesenteric artery thrombosis time (min). Compared with the control group, ^*∗*^*p* < 0.05 and ^*∗∗*^*p* < 0.01; *n* = 6.

The results of the influence on the mesenteric artery thrombosis of mice after administration of YQHX granules are presented in Figure 2. In the blood vessel, the green fluorescent granules represent platelets. Platelets were aggregated in the damaged part, and the thrombosis time was when there was large-area formation of fluorescent granules on the wall. Compared with the control group (21.63 ± 1.56 min), in both the YQHX group and aspirin group, the mesenteric artery thrombosis time damaged by FeCl_3_ could be extended (30.00 ± 1.31 min, *P* < 0.01; 27.67 ± 2.15 min, *P* < 0.05), which could inhibit thrombosis.

### 3.3. Influence of YQHX Granules on the Platelet PAR-1 and MAPK Protein Phosphorylation Levels Induced by THR

**Figure 3:**
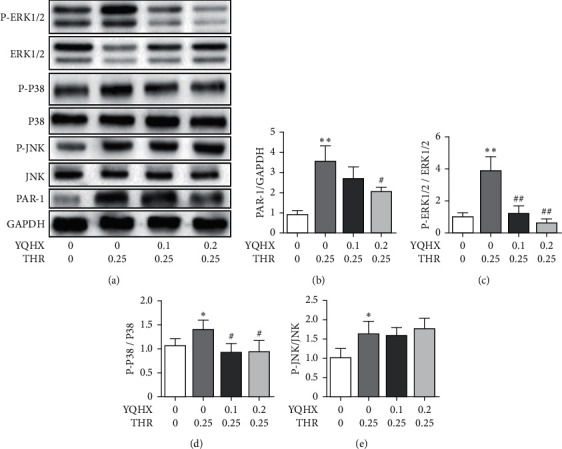
Comparison of the PAR-1, ERK1/2, p38, and JNK protein levels in platelets among various groups. (a) Western blot diagram; (b) PAR-1 protein level; (c) the ERK 1/2 protein level; (d) the P38 protein level; (e) the JNK protein level. Compared with the group with no THR induction, ^*∗*^*p* < 0.05 and ^*∗∗*^*p* < 0.01. Compared with the group with THR induction, ^#^*P* < 0.05 and ^##^*P* < 0.01; *n* = 4.

THR induced platelet to cut the N end of PAR-1 receptor and activate the PAR-1 receptor. See Figure 3 for the results. The PAR-1 protein level increased under the induction of THR, and after intervention using the YQHX granules, the PAR-1 protein level decreased with the increase of YQHX concentration. The growth of MAPK protein phosphorylation level stimulated the aggregation and adhesion functions of platelets, and THR stimulated the increase of the phosphorylation levels of ERK1/2, P38, and JNK. The YQHX granules reduced the phosphorylation levels of ERK1/2 and P38 (*P* < 0.05), but did not have any intervention effect on the phosphorylation level of JNK (*P* > 0.05).

### 3.4. Influence of YQHX Granules on the Intracellular Calcium Level in Platelet-Like Granules Converted from DAMI Cells under the Induction of THR

**Figure 4:**
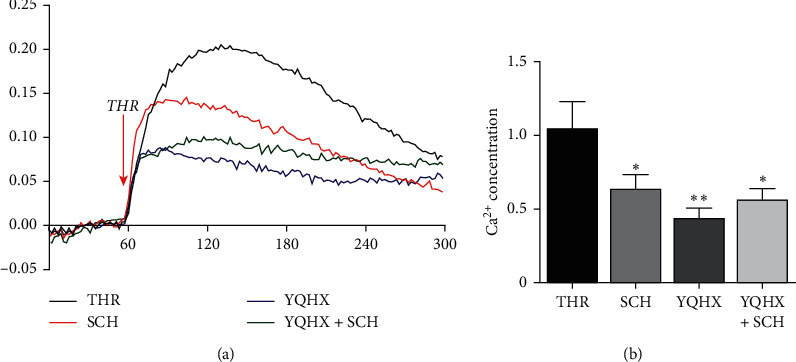
Comparison of intracellular calcium levels of platelet-like granules induced by THR among various groups. (a) Real-time change of intracellular fluorescence after induction by THR for 5 min after coloring with FLUO 4-AM; (b) change of intracellular calcium levels after induction by THR (area under the curve). Compared with the control group (THR induction group), ^*∗*^*p* < 0.05 and ^*∗∗*^*p* < 0.01; *n* = 4.

In this study, PMA was colored using FLUO 4-AM, so as to induce the DAMI cells to convert to platelet-like granules, and after the THR induction, the sum of real-time fluorescence intensity areas under the curve was the calcium level. The results are as shown in Figure 4. In the SCH group, the calcium level induced by THR decreased; compared with the THR group, in the YQHX granule group and mixed intervention group, the intracellular calcium levels declined (*P* < 0.05); however, the difference between the YQHX granule group + SCH group and the YQHX granule group does not have statistical significance (*P* > 0.05).

### 3.5. Influence of YQHX Granules on the PAR-1 and MAPK Protein Phosphorylation Levels in Platelet-Like Granules Converted from DAMI Cells under the Induction of THR

**Figure 5:**
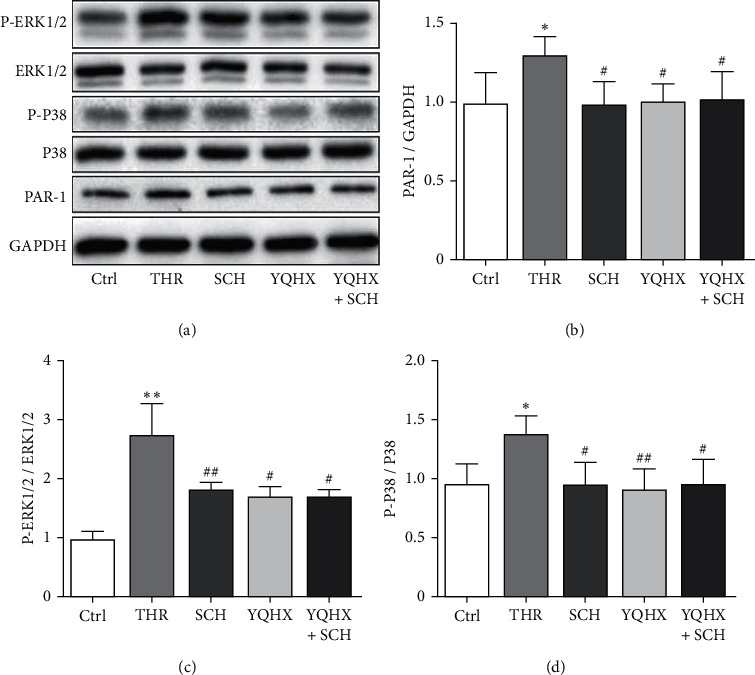
Comparison of the protein levels of PAR-1, ERK1/2, and P38 in platelet-like granules induced by THR among various groups. (a) Western blot diagram; (b) the PAR-1 protein level; (c) the ERK1/2 protein phosphorylation level; (d) the P38 protein phosphorylation level. Compared with the group with no THR induction, ^*∗*^*p* < 0.05 and ^*∗∗*^*p* < 0.01. Compared with the group with THR induction, ^#^*P* < 0.05 and ^##^*P* < 0.01; *n* = 4.

SCH79797 is a selective PAR-1 antagonist, and it was missed with the YQHX granules to observe whether the YQHX granules can work via PAR-1. The results are presented in Figure 5. In the experiment of converting DAMI cells to platelet-like granules, compared with the non-THR induction group, the protein level of PAR-1 and the protein phosphorylation levels of ERK1/2 and P38 were significantly increased in the THR induction group (*P* < 0.05). Compared with the THR induction group, the protein level of PAR-1 and the protein phosphorylation levels of ERK1/2 and P38 were reduced in the SCH group, YQHX granule group, and the YQHX granule + SCH group (*P* < 0.05, *P* < 0.01). By comparing the YQHX granule group and the YQHX granule + SCH group, their difference in the protein phosphorylation levels of PAR-1, ERK1/2, and P38 does not have statistical significance (*P* > 0.05).

## 4. Discussion

When the platelet active substances, such as THR, collagen, ADP, and TXB2, are combined with the platelet membrane receptors, it will trigger transduction of multiple intracellular signals, which will stimulate platelet aggregation and thrombosis [13]. THR is a potent agonist of platelet, which can activate PAR-1 by cutting and hydrolyzing the N end of surface PAR-1 receptor, so as to further stimulate the release of endogenous ADP and P-selectin, thus promoting platelet activation and aggregation [3, 5]. With the activation of PAR-1 receptor, the platelet deformation will be triggered via the G12/13 protein. In the meantime, the Gq protein and inositol triphosphate (IP3) will be activated, which will promote the increase of calcium level and activate the downstream pathway [14]. The results of this study show that when induced by THR, the platelet aggregation rate, the activated PAR-1 protein level in platelets and the platelets converted from DAMI, intracellular calcium level, and MAPK protein phosphorylation level will increase.

Anticoagulant therapy is a basic treatment for cardiovascular diseases, and in clinical treatment, drugs such as aspirin and clopidogrel can inhibit the activation and aggregation of platelets [15]. The traditional Chinese medicines have the effects of benefiting qi for activating blood circulation, and the common traditional Chinese medicines with the effects of benefiting qi for activating blood circulation used in clinical treatment, like Tongxinluo capsule, Taohong Siwu decoction, Yiqi Xinnaoning, and Mailuoning, can prevent platelet activation and inhibit thrombosis [10]. The preliminary data show that YQHX granule can inhibit the plate activation induced by ADP mediated by TXB2 [10]. On the other hand, *Panax ginseng*, *Astragalus membranaceus*, *Radix paeoniae rubra*, *Stigma croci* and other ingredients of YQHX granule also have effects of inhibiting platelet aggregation and antithrombosis. For example, ginsenoside can inhibit the platelet aggregation rate induced by arachidonate, ADP, and collagen [16, 17]. Astragalus membranaceus and total saponins of Astragalus can reduce the platelet aggregation rate and inhibit thrombosis [18, 19]; the blood circulation activating drugs made of Radix paeoniae rubra and *Carthamus tinctorius* have the effects of promoting blood circulation to remove blood stasis, and their extracts benzoic acid, paeoniflorin, and Hydroxysafflor yellow A can also inhibit the platelet aggregation rate [20, 21]. Previous researches on intervention of platelet activation using the traditional Chinese medicine mainly focused on the induction with ADP, collagen, and TXB2. Since THR is a strong agonist of platelet, what about the intervention effects of YQHX granule? Our results show that the YQHX granule can significantly reduce the platelet aggregation rate induced by THR, extend the mouse mesentery thrombosis time, reduce the PAR-1 protein activation level, inhibit the intracellular calcium level in platelet-like granules converted from DAMI cells under the induction of THR, lower the protein phosphorylation levels of ERK1/2 and P38, and inhibit the platelet activation function.

The PAR-1 receptor is the main THR receptor. THR cuts specific sites on the N end of PAR-1 receptor and binds with it, so as to activate the PAR-1 receptor [5]. The activated PAR-1 has specific binding with the G-protein receptors (G12/13, Gq) on the platelet membrane, which activates the intracellular downstream signal transduction pathway and induce platelet activation, and the activated platelets release substances like ADP, adrenaline, and 5-hydroxytryptamine, thus further stimulating the platelet activation [22, 23]. On the other hand, THR converts fibrinogen to fibrous protein, which will promote thrombosis [24]. Therefore, without changing the normal coagulation function of THR, by reducing bleeding complications, the thrombosis rate induced by THR can be reduced by inhibiting the activation of PAR-1 receptor. The results show that the YQHX granule can inhibit the protein activation level of PAR-1 receptor inducted by THR, and the PAR-1 receptor inhibitor SCH79797 can significantly reduce the protein activity of PAR-1. However, the protein activity level of PAR-1 receptor cannot be further reduced by combining the YQHX granule and SCH79797. In other words, compared with the group only using the YQHX granule, the PAR-1 receptor protein cannot be inhibited more effectively by combining the YQHX granule and SCH79797, which implies that the PAR-1 protein is the potential effect target of YQHX granule.

As the second messenger in cells, the calcium ions are indispensable in the activation and aggregation processes of platelets [25]. THR works on the G-protein receptors such as PAR, activates related downstream signal pathway, and triggers change of intracellular calcium level in platelet [26]. From the serine/threonine protein kinases family, MAPKs are the main means to convert the extracellular stimulation to cellular reaction, which are necessary for platelet activation, and the platelet activation can be reduced by inhibiting or removing MAPKs [27]. Previous researches show that the Rk1 derivative G-Rp1 from Radix Ginseng Rubra saponin and *Panax ginseng* saponin can reduce the platelet aggregation induced by THR and collagen; reduce the phosphorylation levels of VASP, ERK2, and P38; and inhibit thrombosis [16, 17]. According to our results, the changes of the intracellular calcium level and the phosphorylation levels of ERK1/2 and P38 during the process of using the YQHX granule to reduce platelet activation were consistent with the findings of previous researches. Similarly, compared with the YQHX granule group, the YQHX granule + SCH79797 joint intervention group did not present difference with statistical significance. This further proves that the YQHX granule inhibits the activation and aggregation of platelets by intervening with the activity of PAR-1 receptor, reducing the intracellular calcium level and the phosphorylation levels of ERK1/2 and P38.

## 5. Conclusion

In conclusion, the YQHX granule can reduce the platelet aggregation rate induced by THR and extend the thrombosis time. Its mechanism is to reduce the platelet activation function induced by THR by inhibiting the activation of PAR-1 signal pathway. Our results have provided the theoretical basis of the TCM treatment for acute coronary syndrome.

## Data Availability

The data used to support the findings of this study are available from the corresponding author upon request.

## Abbreviations

ERK: Extracellular signal regulated kinase
IP3: Inositol triphosphate
MAPK: Mitogen-activated protein kinase
PARs: Protease-activated receptors
PCI: Percutaneous coronary intervention
TCM: Traditional Chinese medicine
THR: Thrombin
TXB2: Thromboxane B2
YQHX: Yiqi Huoxue.
